# Supplementary material for: Epidemiological Characteristics and Antimicrobial Resistance Changes of Carbapenem-Resistant Klebsiella pneumoniae and Acinetobacter baumannii under the COVID-19 Outbreak: An Interrupted Time Series Analysis in a Large Teaching Hospital
Source: Antibiotics (Basel). 2023 Feb 22;12(3):431. doi: 10.3390/antibiotics12030431 (PMC10044178; doi:10.3390/antibiotics12030431)
Supplement: Supplementary file 1 [file antibiotics-12-00431-s001.zip › antibiotics-2061774-supplementary.pdf]

Supplementary Materials

Table S1: The distribution of *A. baumannii*, CRAB, *K. pneumoniae* and CRKP in different groups.

Group		*Acinetobacter baumannii* (n = 2240)		CRAB (n = 1629)		*Klebsiella pneumoniae* (n = 2495)		CRKP (n = 488)	
		n (%[a])	*P*[c]	n (%[b])	*P*	n (%)	*P*	n (%)	*P*
Sex	female	661 (9.45%)	< 0.001	460 (69.59%)	< 0.05	786 (11.24%)	< 0.001	157 (19.97%)	> 0.05
	male	1579 (13.31%)		1169 (74.03%)		1709 (14.41%)		331 (19.37%)	
Age	<1-year-old	81 (29.78%)	< 0.001	39 (48.15%)	< 0.001	43 (15.81%)	< 0.01	0 (0.00%)	< 0.001
	1 to 14-year-old	123 (14.06%)		97 (78.86%)		84 (9.60%)		11 (13.10%)	
	15- to 65-year-old	1261 (10.83%)		938 (74.39%)		1578 (13.55%)		307 (19.46%)	
	> 65-year-old	775 (12.78%)		555 (71.61%)		790 (13.03%)		170 (21.52%)	
Spec-type	sputum	1829 (23.33%)	< 0.001	1373 (75.07%)	< 0.001	1320 (16.83%)	< 0.001	270 (20.45%)	< 0.05
	urine	83 (4.24%)		45 (69.23%)		312 (15.93%)		43 (15.09%)	
	blood	65 (2.25%)		36 (43.37%)		285 (9.87%)		42 (13.64%)	
	drain	17 (2.06%)		18 (72.00%)		93 (11.25%)		18 (21.69%)	
	wound	25 (3.83%)		28 (75.68%)		83 (12.71%)		5 (16.13%)	
	clean midstream urine	37 (8.49%)		7 (63.64%)		31 (7.11%)		25 (26.88%)	
Department	Intensive Care Unit	761 (27.34%)	< 0.001	657 (86.33%)	< 0.001	396 (14.23%)	< 0.01	129 (32.58%)	< 0.001
	Rehabilitation Center	335 (11.10%)		282 (84.18%)		419 (13.89%)		129 (30.79%)	
	Neurology	349 (21.07%)		261 (74.79%)		222 (13.41%)		41 (18.47%)	
	Infectious Diseases	77 (5.90%)		27 (35.06%)		254 (19.45%)		32 (12.60%)	
	Surgery	62 (4.91%)		18 (29.03%)		171 (13.53%)		28 (16.37%)	
	Emergency Department	62 (6.78%)		39 (62.90%)		125 (13.68%)		11 (8.80%)	
	Neonatology	114 (32.39%)		63 (55.26%)		69 (19.60%)		1 (1.45%)	
Wards	in	2211 (12.47%)	< 0.001	1611 (72.86%)	> 0.05	2377 (13.41%)	< 0.01	476 (20.03%)	< 0.01
	out	29 (2.57%)		18 (62.07%)		118 (10.46%)		12 (10.17%)	

Note. [a]% refers to percentage of pathogenic bacteria in different groups, which was the number of detected specific pathogenic bacteria/the number of detected all pathogenic bacteria in the same period ×100%. [b]% refers to the percentage of isolates resistant in different groups, which was the number of resistant isolates of a

specific pathogen in the population/the total number of specific pathogens detected in the population ×100%. [c] P values were calculated by 2 test. $P < 0.05$ is considered statistically significant.

Table S2. Results of the Segmented Regression Analysis of number of *A. baumannii*, *K. pneumoniae* and the CRAB, CRKP resistance rate after the removal of duplicate isolates

**Outcomes**	**Coefficient**	**Standard Error**	***t* (*z*)**	***P*-value**
Number of *Acinetobacter baumannii* detected (DW=1.384)				
Baseline level (β_0)	3.159	0.064	49.168	< 0.001
Baseline trend (β_1)	-0.008	0.002	-3.375	< 0.001
Level change after COVID-19 (β_2)	-0.359	0.219	-1.640	0.101
**Trend change after COVID-19 (β_3)**	**0.008**	**0.004**	**2.069**	**< 0.05**
Resistance rate of CRAB (DW=1.773)				
Baseline level (β_0)	0.729	0.040	18.075	< 0.001
Baseline trend (β_1)	-0.002	0.001	-1.494	0.1388
**Level change after COVID-19 (β_2)**	**0.455**	**0.121**	**3.757**	**< 0.001**
**Trend change after COVID-19 (β_3)**	**-0.006**	**0.002**	**-2.564**	**< 0.05**
Number of *Klebsiella pneumoniae* detected (DW=1.807)				
Baseline level (β_0)	3.244	0.060	54.119	< 0.001
Baseline trend (β_1)	-0.004	0.002	-1.736	0.083
**Level change after COVID-19 (β_2)**	**-1.373**	**0.193**	**-7.110**	**< 0.001**
**Trend change after COVID-19 (β_3)**	**0.022**	**0.003**	**6.526**	**< 0.001**
Resistance rate of CRKP (DW=1.963)				
Baseline level (β_0)	0.101	0.036	2.791	< 0.05
Baseline trend (β_1)	0.002	0.001	1.846	0.068
Level change after COVID-19 (β_2)	-0.031	0.137	-0.229	0.819
Trend change after COVID-19 (β_3)	-0.001	0.002	-0.278	0.782

Note. DW, Durbin-Watson coefficient. β_2 and β_3 represent the change in the level and trend of the outcome indicators (compared to β_0 and β_1) since the start of COVID-19. P value of pathogenic bacteria was calculated by Z test. P value of isolates resistance was calculated by t-test. P < 0.05 is considered statistically significant. Bold text represents significant results after the COVID-19 outbreak.

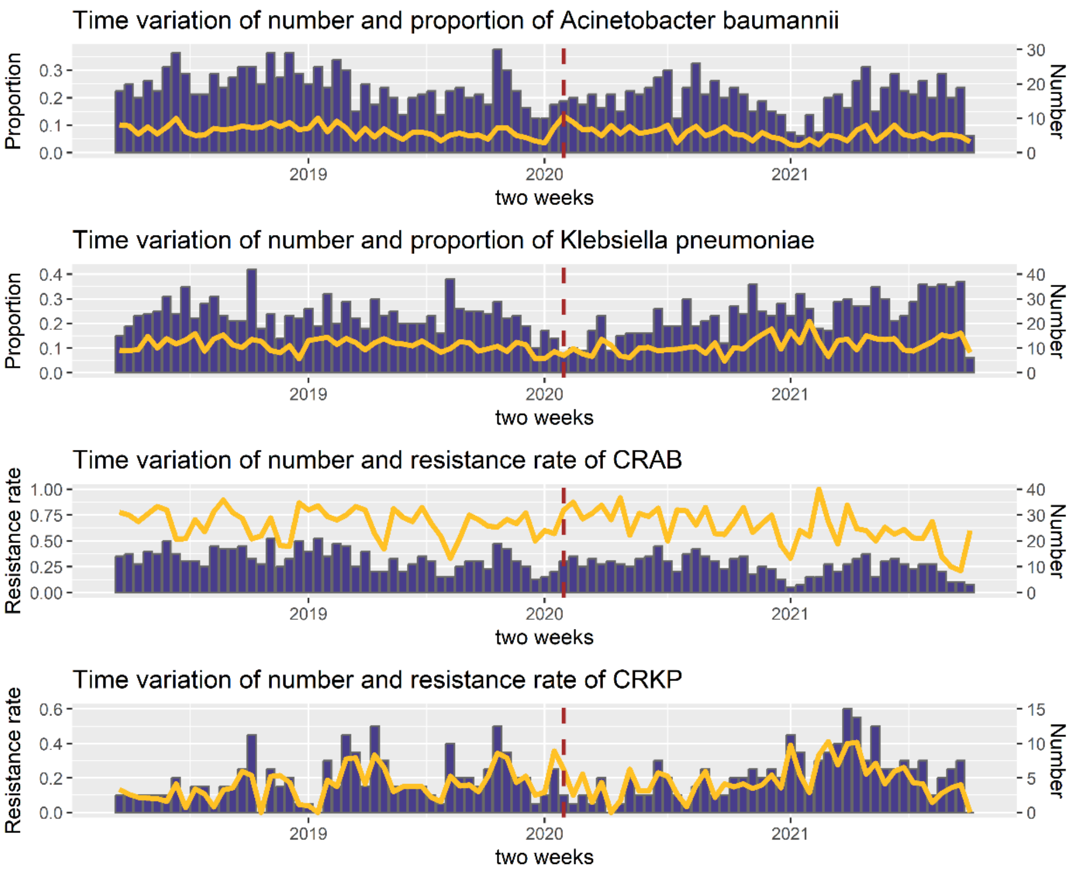

Figure S1. Time trends of pathogens and drug-resistant bacteria after removing duplicates. Data were collected on a biweekly basis. The dark red dashed line indicates the COVID-19 outbreak. The yellow line represents the proportion of the pathogen or resistance rate of the drug-resistant bacteria. The blue bars show the number of pathogenic or drug-resistant bacteria.
